# Laparoscopic duodenojejunostomy for recurrent acute pancreatitis associated with superior mesenteric artery syndrome in a medically complex patient: A case report and literature review

**DOI:** 10.1097/MD.0000000000049601

**Published:** 2026-07-03

**Authors:** Takayuki Hirano, Keita Kogure, Yosuke Watanabe, Manabu Hanada

**Affiliations:** a Department of Pediatric Surgery, Tokyo Metropolitan Ohtsuka Hospital, Tokyo, Japan; b Department of Pediatric Surgery, Nihon University School of Medicine, Tokyo, Japan.

**Keywords:** acute pancreatitis, case report, duodenojejunostomy, omega-loop reconstruction, superior mesenteric artery syndrome

## Abstract

**Rationale:**

Superior mesenteric artery syndrome (SMAS) is a rare cause of duodenal obstruction, and acute pancreatitis associated with SMAS is extremely uncommon. The causal relationship is usually inferred from persistent duodenal obstruction, exclusion of other etiologies, and resolution after decompression.

**Patient concerns:**

A 19-year-old male with a history of esophageal atresia repair, previous upper abdominal surgery, refractory epilepsy, severe developmental delay, and a bedridden status presented with recurrent vomiting and acute pancreatitis. Pancreatitis recurred over a period of 4 years despite repeated conservative management and eventually increased to approximately once per month.

**Diagnoses:**

Contrast-enhanced computed tomography revealed marked gastric and duodenal dilation, compression of the third portion of the duodenum between the aorta and the superior mesenteric artery, and a narrowed aortomesenteric angle of 18°. Other causes of pancreatitis, including gallstones, pancreaticobiliary maljunction, and infection, were excluded. Recurrent acute pancreatitis associated with SMAS-related duodenal obstruction was diagnosed.

**Interventions:**

Conservative treatment included bowel rest, nasogastric decompression, intravenous fluid and electrolyte correction, and prokinetic medication. Because recurrent pancreatitis persisted, laparoscopic duodenojejunostomy with omega-loop reconstruction was performed at age 23. Dense intra-abdominal adhesions precluded safe intracorporeal anastomosis; therefore, extracorporeal side-to-side duodenojejunostomy was completed.

**Outcomes:**

Postoperative contrast studies confirmed unobstructed passage through the anastomosis, with no leakage or residual obstruction. During 3 years of follow-up, no pancreatitis recurrence, bile reflux, or readmission occurred, and the patient’s weight increased.

**Lessons:**

Although causality cannot be definitively established from a single case, surgical duodenal decompression may be considered in selected patients with recurrent pancreatitis associated with persistent SMAS-related duodenal obstruction after the exclusion of alternative causes and failure of conservative management. Continued long-term surveillance is required after omega-loop reconstruction.

## 1. Introduction

Superior mesenteric artery syndrome (SMAS) is characterized by compression of the third portion of the duodenum between the superior mesenteric artery and the aorta, resulting in proximal duodenal obstruction.^[1]^ Although vomiting, abdominal pain, and weight loss are common manifestations, acute pancreatitis secondary to SMAS is extremely rare. The causal mechanism is not always directly demonstrable, but it has been hypothesized that duodenal obstruction increases intraluminal pressure around the papilla of Vater, resulting in impaired pancreaticobiliary drainage and reflux of duodenal contents into the pancreatic duct.^[1]^

Surgical treatment is generally reserved for patients with persistent or recurrent symptoms despite conservative management. Duodenojejunostomy is regarded as a reliable decompressive procedure, but the optimal approach and reconstruction method may vary according to the patient’s condition, operative history, and the risk of postoperative reflux.^[1,2]^ In particular, evidence regarding SMAS-associated recurrent acute pancreatitis is limited to isolated case reports.

Herein, we report a case of recurrent acute pancreatitis associated with SMAS in a medically complex patient with a history of multiple prior surgeries and severe neurological impairment, in whom laparoscopic duodenojejunostomy was performed with favorable outcomes. This report includes a literature review discussing the inferential basis for the diagnosis, the conservative management attempted before surgery, the rationale for selecting omega-loop reconstruction despite severe adhesions, and the 3-year postoperative follow-up.

## 2. Case report

### 2.1. Patient information

The patient was a 19-year-old male with a history of congenital esophageal atresia (Gross type C) repaired in infancy, and he had been followed in our department since birth. He also had refractory epilepsy, severe developmental delay, and a bedridden status. His previous surgical history included a Nissen-type fundoplication (Boerema-Filler method) for gastroesophageal reflux disease at 2 years of age, adhesive ileus after surgery, and appendectomy for appendicitis with peritonitis at 8 years of age.

### 2.2. Clinical findings

**Figure 1.**
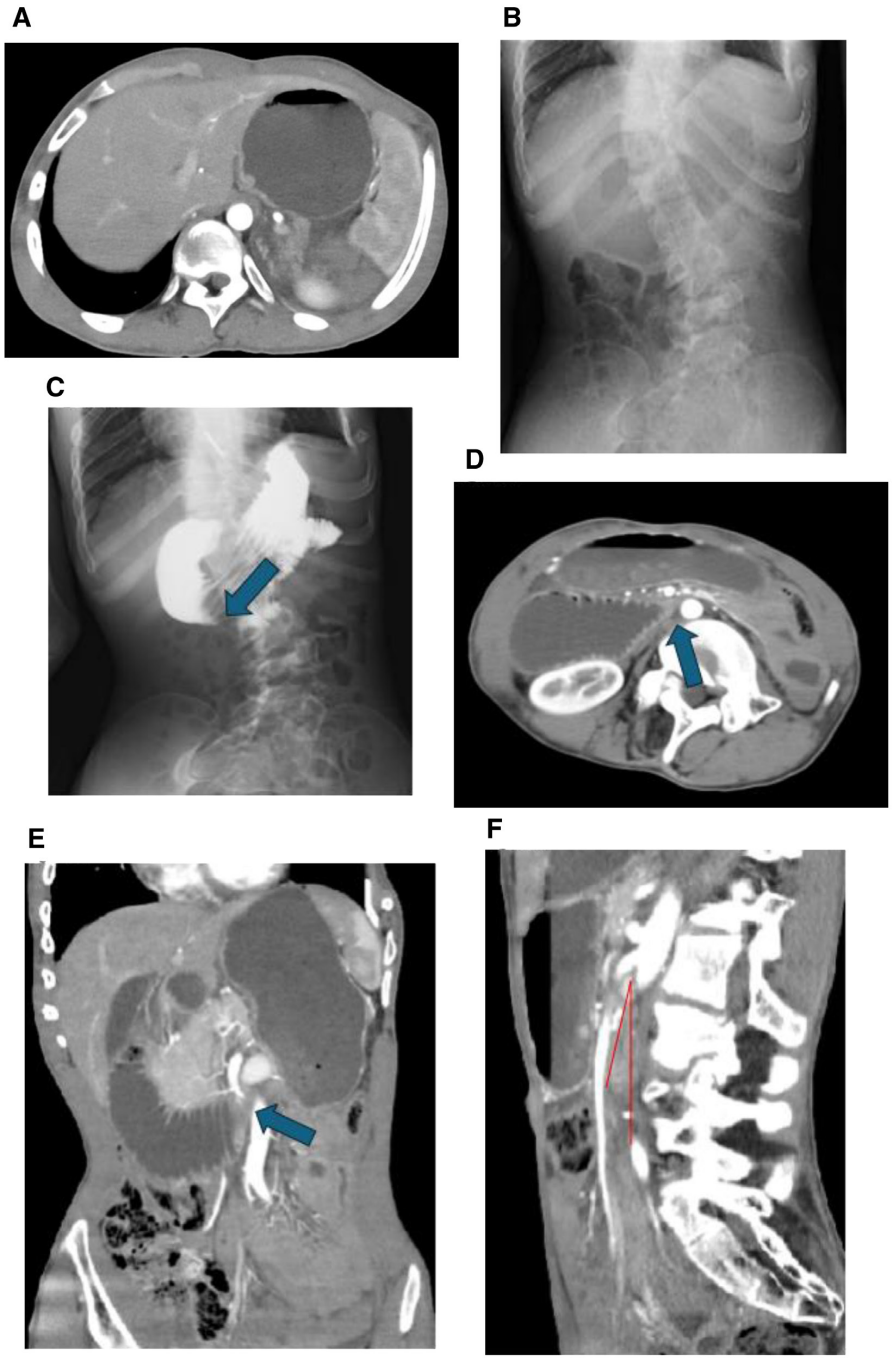
Clinical and radiological findings of the patient. (A) Abdominal contrast-enhanced CT showed increased fat stranding around the pancreatic body and tail. (B) Standing plain abdominal X-ray revealed dilation of the stomach and duodenum. (C) During an upper gastrointestinal contrast study performed in the standing position, the passage of contrast medium from the stomach into the duodenum was smooth; however, a stricture was observed in the horizontal portion of the duodenum, and the contrast medium did not flow beyond that point. (D, E) Axial and coronal views of contrast-enhanced abdominal CT demonstrated dilation of the stomach and the horizontal duodenum compressed between the superior mesenteric artery and the aorta (arrow, beak sign). (F) In the sagittal view, the aortomesenteric angle was narrowed to 18°, compared with the normal angle of approximately ≥22°. CT = computed tomography.

At 19 years of age, he presented with recurrent vomiting. Laboratory tests showed serum amylase and lipase levels of 1348 U/L and 202 IU/L, respectively, and contrast-enhanced abdominal computed tomography (CT) revealed inflammatory changes around the pancreatic body and tail (Fig. 1A). Consequently, the patient was diagnosed with acute pancreatitis. A timeline of the clinical course is presented in Table 1.

**Table 1:** Timeline of clinical events.

Time point	Clinical event
Infancy	Congenital esophageal atresia (Gross type C) was repaired.
2 yr of age	Nissen-type fundoplication (Boerema-Filler method) was performed for gastroesophageal reflux disease.
8 yr of age	Appendectomy was performed for appendicitis with peritonitis; postoperative adhesive ileus also occurred.
19 yr of age	The patient presented with recurrent vomiting and was diagnosed with acute pancreatitis and SMAS-related duodenal obstruction.
19–23 yr of age	Recurrent pancreatitis occurred despite repeated conservative management; the frequency gradually increased.
23 yr of age	Pancreatitis recurred approximately once per month, and laparoscopic duodenojejunostomy with omega-loop reconstruction was performed.
Postoperative day 7	Upper gastrointestinal series confirmed smooth passage through the duodenojejunal anastomosis without leakage or residual obstruction.
Postoperative day 11	The patient was discharged home after an uneventful postoperative course.
3-yr follow-up	No recurrence of pancreatitis, no readmission, and no vomiting suspected to be due to bile reflux were observed; the patient’s weight began to increase.

SMAS = superior mesenteric artery syndrome.

### 2.3. Diagnostic assessment

A plain abdominal X-ray revealed dilation of the stomach and duodenum (Fig. 1B). An upper gastrointestinal contrast study demonstrated narrowing of the horizontal portion of the duodenum (Fig. 1C). Contrast-enhanced abdominal CT showed marked dilation of the stomach and proximal duodenum, a beak-like transition at the third portion of the duodenum compressed between the aorta and the superior mesenteric artery (Fig. 1D and E), and narrowing of the aortomesenteric angle to 18° (Fig. 1F). Based on these findings, the patient was diagnosed with SMAS. Because the patient had a congenital anomaly and recurrent pancreatitis, biliary and pancreaticobiliary causes were carefully evaluated. Abdominal ultrasound and contrast-enhanced CT scans ruled out gallstones, abnormalities at the pancreaticobiliary junction, congenital biliary dilation, and infection as alternative causes of pancreatitis.

### 2.4. Therapeutic intervention

Based on these findings, the patient was diagnosed with recurrent acute pancreatitis most likely associated with SMAS-related duodenal obstruction. During the initial admission, conservative treatment consisted of bowel rest, decompression via a nasogastric tube, intravenous fluid and electrolyte correction, and continuation of oral prokinetic medication. The patient’s symptoms and laboratory findings improved rapidly. Oral food intake was resumed after a 3-day fasting period, and the patient was discharged home on the 10th hospital day. Parenteral nutrition or postpyloric enteral tube feeding was not introduced at that time because obstructive symptoms resolved promptly, and oral intake was tolerated during stable periods. During subsequent hospitalizations, the patient was discharged home within a week after employing similar conservative regimens. Regular postural therapy was difficult to maintain at home because of the patient’s bedridden status and the caregiver burden. However, the frequency of recurrence gradually increased, reaching approximately once a month by the time the patient was 23 years old. Because pancreatitis became frequent despite repeated conservative management and imaging studies continuously showed SMAS-related duodenal obstruction, prolonged nutritional therapy was considered insufficient as definitive treatment, and surgical decompression was selected.

**Figure 2.**
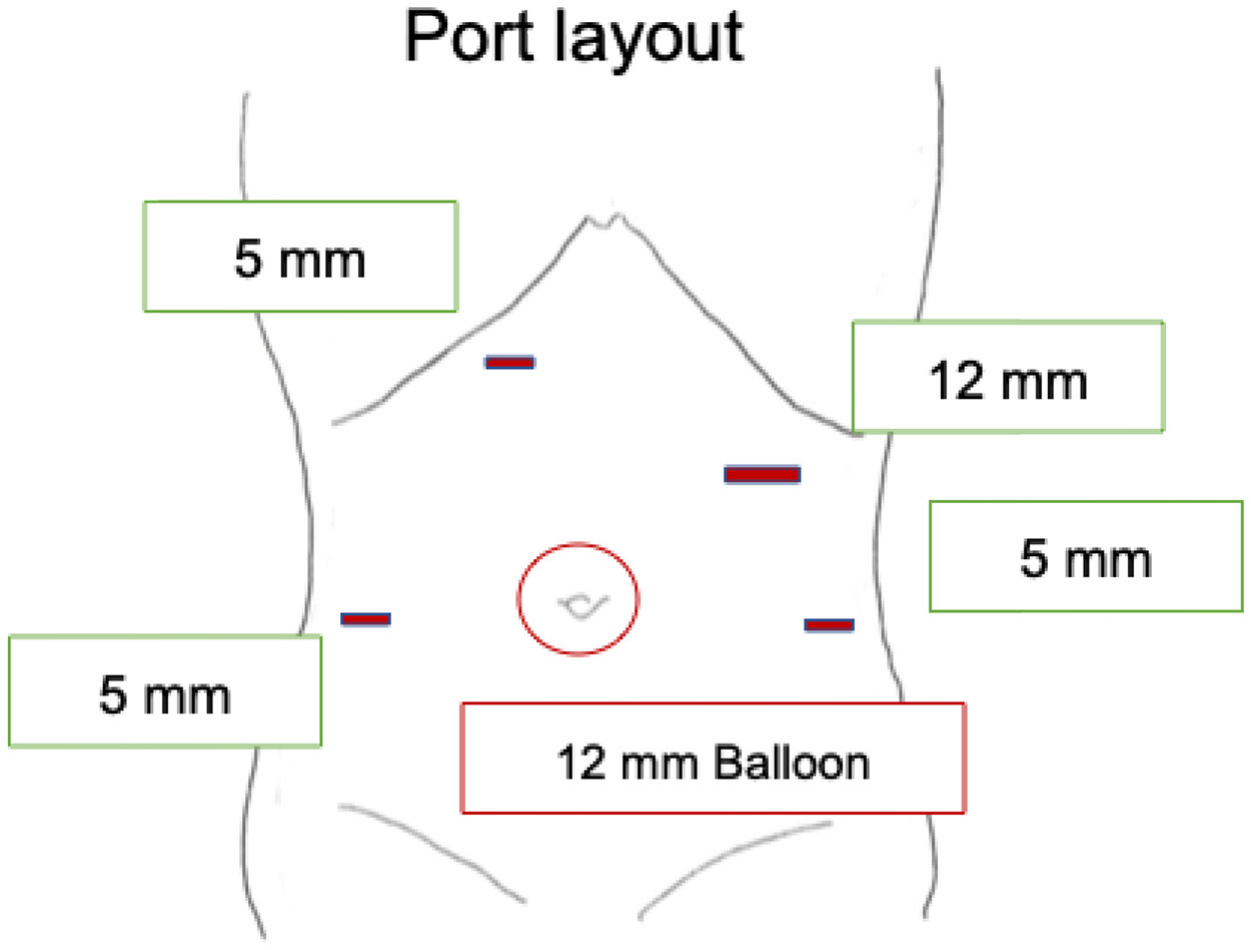
Port placement: 12-mm free access port at the umbilicus, 12-mm port in the left upper abdomen, and 5-mm ports at the epigastrium and both lateral abdomens at the umbilical level.

**Figure 3.**
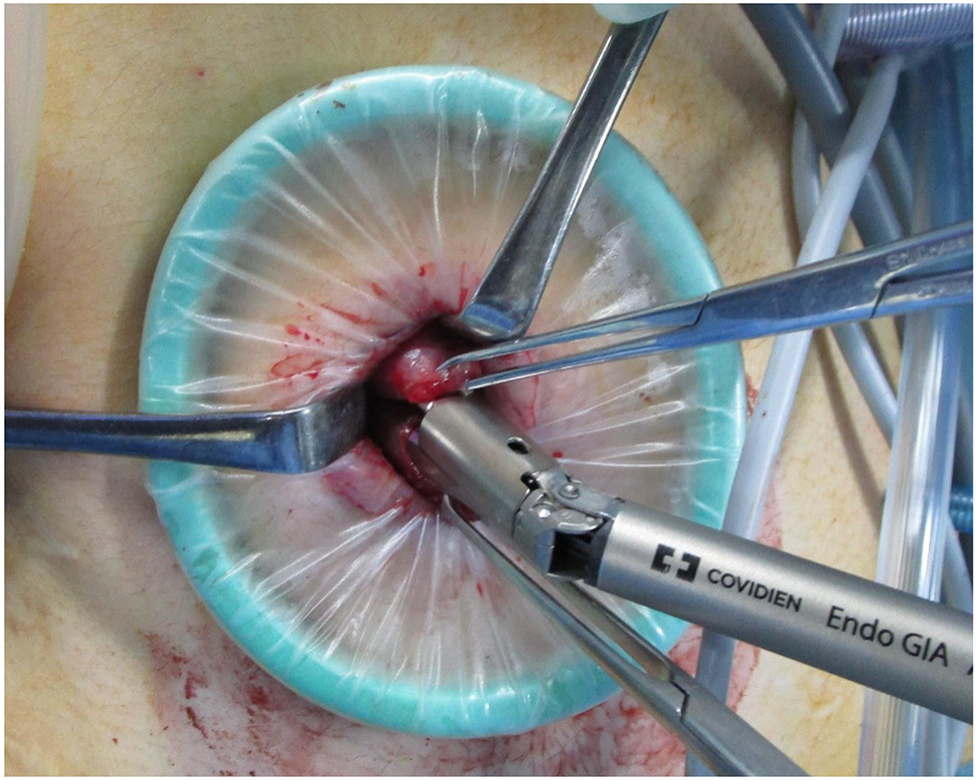
Intracorporeal anastomosis was not feasible because of dense intra-abdominal adhesions. Therefore, extracorporeal side-to-side duodenojejunostomy was performed using a stapling device, and the enterotomy was closed with continuous absorbable sutures.

A laparoscopic duodenojejunal anastomosis was performed using a 5-port technique (Fig. 2). The port placement consisted of a 12-mm port at the umbilicus, a 12-mm port in the left upper abdomen, and 5-mm ports bilaterally in the epigastric region and at the umbilical level. Under laparoscopic visualization, severe adhesions between the stomach, liver, and abdominal wall were dissected. The duodenum was mobilized using the Kocher maneuver. A jejunal loop located 30 cm distal to the ligament of Treitz was identified. Owing to severe adhesions, safe intra-abdominal anastomosis was difficult; therefore, an extra-abdominal bypass procedure using omega-loop reconstruction was chosen. A side-to-side duodenojejunal anastomosis was performed using an automatic stapler (Fig. 3), and the enterotomy was closed with continuous absorbable sutures.

### 2.5. Follow-up and outcomes

**Figure 4.**
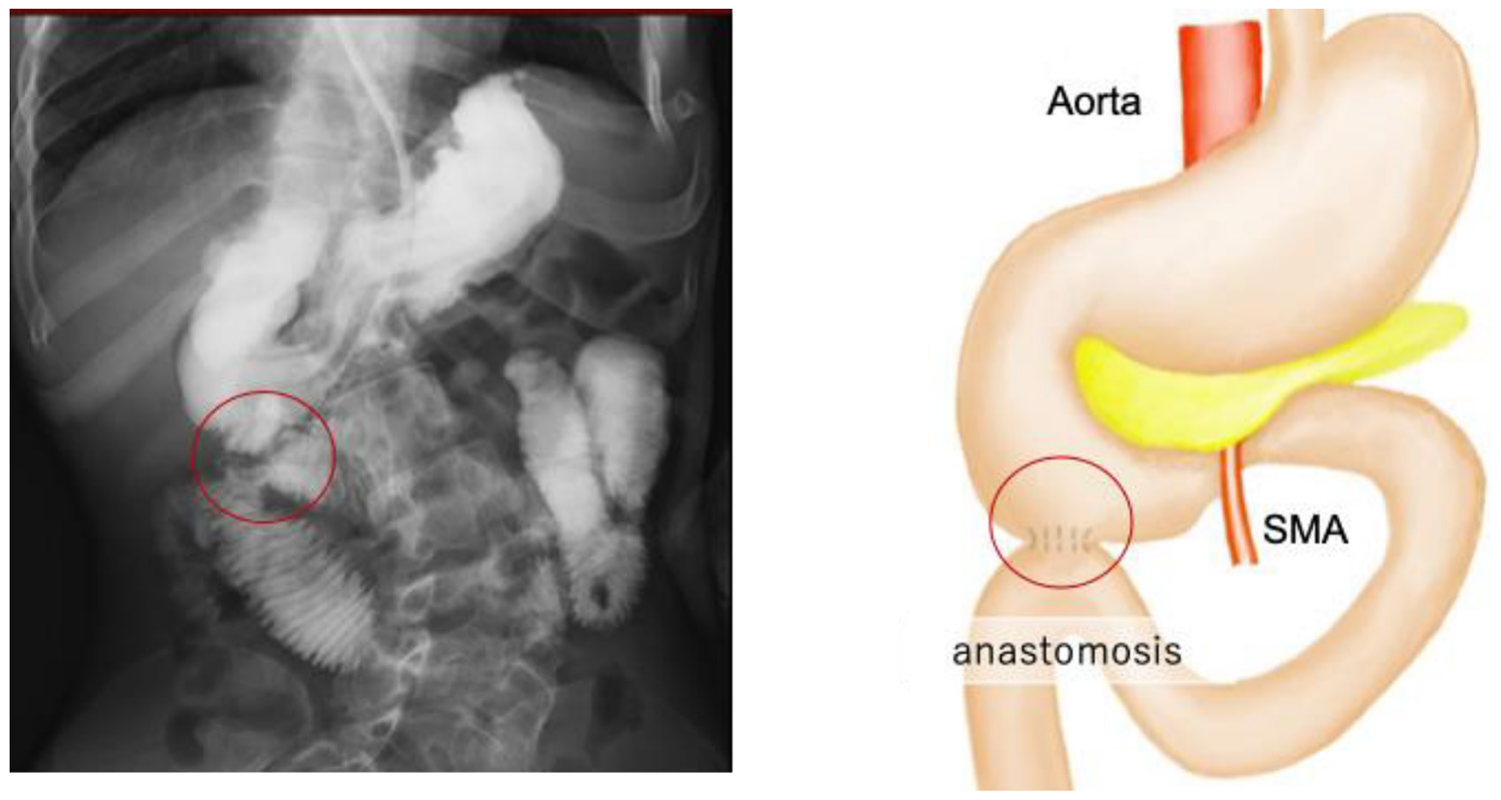
A postoperative upper gastrointestinal contrast study performed on day 7 demonstrated smooth passage of the contrast medium through the stomach, duodenum, and anastomosed jejunum. No anastomotic leakage was observed. SMA = superior mesenteric artery.

The postoperative course was uneventful, and serum amylase and lipase levels rapidly returned to normal. An upper gastrointestinal series performed on the seventh postoperative day confirmed that contrast medium flowed smoothly through the duodenojejunal anastomosis, with no evidence of anastomotic leakage or residual obstruction (Fig. 4). Oral intake was resumed immediately, and the patient was discharged home on the 11th postoperative day. The patient’s condition remained stable after discharge, and his weight began to increase. During the 3-year follow-up period, no recurrence of pancreatitis was observed, and no readmissions for any reason occurred, including vomiting suspected to be due to bile reflux or recurrence of pancreatitis.

## 3. Discussion

SMAS is rare, with an estimated incidence of 0.013% to 0.3%.^[1]^ It occurs when the aortomesenteric angle becomes narrower than 25° (normal, 38°–65°). The causes include anatomical abnormalities and severe weight loss, leading to the loss of mesenteric fat tissue, as observed in conditions such as anorexia nervosa or malabsorption syndromes. Clinical presentation varies from acute duodenal obstruction to chronic recurrent symptoms, including abdominal pain and vomiting. Imaging studies, particularly CT and magnetic resonance angiography, are useful for evaluating duodenal obstruction and measuring the aortomesenteric angle.^[2]^

This case has 3 educational features. First, recurrent acute pancreatitis was associated with radiologically evident SMAS after the exclusion of common alternative causes. Second, the case involved multiple factors that complicated both the pathogenesis and management of SMAS, including severe intra-abdominal adhesions from previous operations, severe neurological impairment, a bedridden status, and nutritional vulnerability. Third, pancreatitis did not recur for up to 3 years after surgical duodenal decompression. This supports the clinical relevance of the obstructive mechanism, although it does not prove causality and underscores the need for longer follow-up.

The causal relationship between SMAS and pancreatitis remains inferential in most reported cases. Anatomically, obstruction at the third portion of the duodenum can cause persistent dilation of the stomach and proximal duodenum. This may increase intraduodenal pressure near the ampulla of Vater, impair the anti-reflux function of the sphincter of Oddi, and promote reflux of duodenal contents into the pancreaticobiliary ducts.^[1]^ Pancreatic duct hypertension and impaired drainage may then trigger acute pancreatitis. In the present case, the diagnosis was supported by recurrent vomiting and radiographic duodenal obstruction; a markedly narrowed aortomesenteric angle; exclusion of apparent biliary, infectious, and structural pancreaticobiliary causes, including gallstones and pancreaticobiliary maljunction; recurrent pancreatitis despite initially successful conservative treatment; and absence of pancreatitis recurrence after duodenal decompression. Combined, these findings support SMAS-related obstruction as the most plausible explanation. However, because direct demonstration of duodenopancreatic reflux was not possible, a causal relationship should be interpreted as highly suggestive rather than definitive.

Previously reported surgical cases of pancreatitis associated with SMAS are summarized in Table 2.^[3–9]^ Although the number of reported cases is small, most patients underwent a form of duodenal bypass and had no reported recurrence of pancreatitis after surgery. Because many of the cases are older, only a limited number of laparoscopic surgical cases have been described, and postoperative follow-up has often been short or incompletely reported. This case contributes to the existing literature by demonstrating a 4-year history of recurrent pancreatitis, an increase in the frequency of attacks despite conservative treatment, a technically challenging surgical procedure after multiple prior abdominal surgeries, and the absence of pancreatitis recurrence during a 3-year postoperative follow-up, which is longer than that reported in most previously reported cases (Table 2).

**Table 2:** Reported surgical cases of pancreatitis associated with superior mesenteric artery syndrome and their outcomes.

Year/author	Age/sex	Background	Procedure/approach	Conservative treatment before surgery	Follow-up/pancreatitis recurrence
1966/Kelly et al^[3]^	40/F	None reported	Duodenojejunostomy/open	Not reported	Not reported/no recurrence reported
1976/Feiss et al^[4]^	20/F	Post-laminectomy	Duodenojejunostomy/open	Nasogastric suction, bowel rest, and intravenous fluids	9 mo/no recurrence reported
1988/Ammaturo et al^[5]^	58/F	None reported	Duodenojejunostomy/open	Not reported	10 months/no recurrence reported
2006/Arbell et al^[6]^	16/M	Cerebral palsy	Duodenoduodenostomy/open	Enteral and parenteral alimentation	Not specified
2009/Petrosyan et al^[7]^	54/F	After Nissen fundoplication	Duodenojejunostomy/open	Not reported	1 yr/no recurrence reported
2011/Alnabulsi et al^[8]^	24/F	Weight loss	Omega-loop duodenojejunostomy with Braun anastomosis/laparoscopic	Not reported	Not reported/no recurrence reported
2022/Hsu et al^[9]^	17/not stated	Neurogenic dysphagia, weight loss	Duodenojejunostomy/laparoscopic	Bowel rest and intravenous fluids	Not reported/no recurrence reported
Present case	23/M	Post-esophageal atresia repair and fundoplication, severe neurological impairment	Omega-loop duodenojejunostomy/laparoscopic-assisted extracorporeal anastomosis	Nasogastric suction, bowel rest, and intravenous fluids	3 yr/no recurrence

Conservative therapy is the first-line treatment for SMAS and generally includes gastric decompression, correction of fluid and electrolyte abnormalities, nutritional support, postural therapy, and prokinetic agents.^[2]^

One approach to nutritional management involves central venous nutrition or continuous enteral feeding via a nasojejunal tube placed beyond the site of obstruction to promote weight gain, with the aim of improving the aortomesenteric angle.^[1]^ In the present case, the patient initially responded to bowel rest, nasogastric decompression, intravenous fluids, electrolyte correction, and prokinetic therapy during each acute episode. Because oral intake could be resumed within several days and stable periods allowed oral feeding, parenteral nutrition and continuous nasojejunal feeding were not selected at the initial stage. Regular postural therapy was also difficult to maintain at home because of the patient’s bedridden status and caregiver burden. When pancreatitis recurred monthly despite this approach and imaging showed persistent obstruction, prolonged tube feeding and central venous nutrition were considered; however, these were not chosen as definitive treatments because they would not have immediately resolved the obstructive mechanism and would have increased the burden of home care. These considerations supported the decision to proceed with surgical decompression.

The decision to operate was not based solely on the diagnosis of SMAS, but on the progression from intermittent episodes to frequent recurrent pancreatitis and the persistence of an obstructive anatomical mechanism. This point is clinically important because recurrent pancreatitis may represent not only a complication of SMAS but also an indication that decompression should be considered when conservative management no longer prevents recurrence.

The patient’s background requires careful interpretation. A direct causal relationship between repaired esophageal atresia and SMAS cannot be established based on a single case. However, the patient’s history of multiple surgeries, including radical esophageal atresia repair and subsequent intra-abdominal adhesions, clearly influenced operative planning. In addition, severe neurological impairment and the patient’s bedridden status may have contributed to nutritional vulnerability and reduced mobility, both of which may worsen or maintain SMAS. Recent pediatric laparoscopic duodenojejunostomy series also emphasized that comorbid gastrointestinal, psychiatric, connective tissue, or developmental conditions may contribute to SMAS development and complicate recovery.^[10,11]^ Thus, the present case is best interpreted not as evidence that esophageal atresia causes SMAS, but as an example of SMAS-associated pancreatitis in a medically complex patient in whom comorbidity affected both management and surgical strategy. For this reason, the findings should be extrapolated to patients with uncomplicated SMAS with caution.

When diagnosing recurrent pancreatitis, it is important to exclude pancreaticobiliary maljunction, which is a congenital anomaly of the pancreaticobiliary junction and can be associated with pancreatitis through pancreaticobiliary reflux. It is also clinically important because it has been associated with a later risk of biliary cancer, particularly gallbladder cancer.^[12]^

In this case, abdominal ultrasound and contrast-enhanced CT scans revealed no gallstones, pancreatic duct dilation, bile duct dilation, or other findings suggestive of pancreaticobiliary anomalies.

Several surgical options have been described for SMAS. Strong’s procedure avoids intestinal anastomosis by dividing the ligament of Treitz; however, this method may be less reliable when the duodenum remains fixed or when dense adhesions limit mobilization. Gastrojejunostomy is technically simple but does not directly decompress the obstructed duodenum and may be associated with blind-loop symptoms or persistent duodenal stasis. Duodenojejunostomy directly bypasses the obstructed segment and is currently the most widely accepted surgical option when conservative therapy fails.^[1,2]^

The reconstruction method should be individualized. Roux-en-Y reconstruction may reduce bile reflux into the duodenum and stomach, but it requires additional bowel division and anastomosis, increases operative complexity, and carries a risk of internal hernia. In contrast, omega-loop duodenojejunostomy is technically simpler and may reduce operative manipulation and time, which was advantageous in this patient because of severe adhesions and medical frailty. A trade-off is the potential risk of bile reflux, anastomotic ulceration, or anastomotic stricture.^[8,13–15]^ Therefore, the choice of omega-loop reconstruction should be presented as a pragmatic balance between technical feasibility and long-term reflux risk, rather than as a universally preferred method.

Laparoscopy offers potential advantages, including reduced wound morbidity, earlier recovery, and improved visualization of the upper abdomen; several adult and pediatric series support laparoscopic duodenojejunostomy as a feasible option in selected patients.^[10,11,16]^ However, this approach can be challenging in patients with multiple previous abdominal operations. In the present case, laparoscopy permitted adhesiolysis and assessment of the operative field, but severe adhesions required an extracorporeal rather than intracorporeal anastomosis. This technical modification may be useful in similar patients with hostile abdomens.

Long-term follow-up is essential. Three years without pancreatitis recurrence is clinically significant; however, this period is insufficient to exclude late complications of duodenojejunostomy or omega-loop reconstruction. Recent long-term data after laparoscopic duodenojejunostomy for SMAS suggest that symptoms and body mass index may improve; however, patients with concurrent gastrointestinal motility disorders may suffer from persistent symptoms or require additional interventions.^[16]^ This is particularly relevant to medically complex or neurologically impaired patients. Therefore, postoperative assessment should include pancreatitis recurrence, nutritional status, vomiting or abdominal pain, symptoms suggestive of bile reflux, anastomotic patency, and caregiver-observed quality of life when formal patient-reported assessment is not feasible. Furthermore, because recurrent acute pancreatitis has been linked to an increased long-term risk of cancer, these findings underscore the need for longer-term follow-up.^[17]^

This report has several limitations. First, as a single-case report, it cannot definitively establish a causal relationship between SMAS and pancreatitis. Second, no direct evidence of duodenopancreatic duct reflux was obtained. Third, although the patient was treated conservatively, the nutritional strategy was limited because symptoms initially improved rapidly and oral food intake was possible during stable periods; therefore, the relative efficacy of more intensive nutritional therapy cannot be assessed. Fourth, the patient’s medically complex background, including previous foregut surgery, severe neurological impairment, a bedridden status, and severe adhesions, limits the generalizability of the findings to patients with uncomplicated SMAS. Fifth, although the 3-year course without recurrence of pancreatitis is promising, longer-term follow-up is necessary to evaluate late complications such as bile reflux, anastomotic ulcers, anastomotic strictures, and recurrence of obstructive symptoms.

## 4. Conclusion

Acute pancreatitis associated with SMAS is rare and should be considered when recurrent pancreatitis coexists with persistent proximal duodenal obstruction and no other apparent cause is identified. Although causality cannot be definitively proven based on a single case, the absence of recurrence after decompression supports SMAS-related obstruction as the most plausible mechanism in this patient. This case suggests that surgical duodenal decompression may be an option in selected medically complex patients when conservative treatment, including decompressive and nutritional measures, fails to prevent recurrent pancreatitis. However, this experience should be generalized cautiously, and the benefit of omega-loop reconstruction should be balanced against the possibility of late reflux-related or anastomotic complications. Continued long-term follow-up is warranted.

## 5. Patient perspective

The patient was unable to provide his perspective because of severe developmental delay. Therefore, formal patient-reported outcomes could not be obtained; postoperative assessment was based on clinical follow-up and caregiver reports.

## Acknowledgments

We would like to thank Editage (www.editage.jp) for English-language editing.

## Author Contributions

**Conceptualization:** Takayuki Hirano.

**Data curation:** Keita Kogure.

**Investigation:** Takayuki Hirano.

**Validation:** Takayuki Hirano, Manabu Hanada.

**Visualization:** Takayuki Hirano.

**Resources:** Keita Kogure.

**Supervision:** Yosuke Watanabe, Manabu Hanada.

**Writing – original draft:** Takayuki Hirano.

**Writing – review & editing:** Takayuki Hirano, Keita Kogure, Yosuke Watanabe, Manabu Hanada.
